# Supplementary material for: Phylogenetic clustering of microbial communities as a biomarker for chemical pollution
Source: FEMS Microbiol Ecol. 2025 May 2;101(5):fiaf047. doi: 10.1093/femsec/fiaf047 (PMC12067926; doi:10.1093/femsec/fiaf047)
Supplement: fiaf047_Supplemental_Files [file fiaf047_supplemental_files.zip › supplementary-information.pdf]

Supplementary Information for: Phylogenetic clustering of microbial communities as a biomarker for chemical pollution

Thomas P. Smith[1], Rachel Hope[2], Thomas Bell[1]

[1] The Georgina Mace Centre for the Living Planet, Department of Life Sciences, Silwood Park Campus, Imperial College London, Ascot, UK.
[2] Department of Fundamental Microbiology, Faculty of Biology and Medicine, University of Lausanne, Lausanne, Switzerland.

Supplementary Figures:

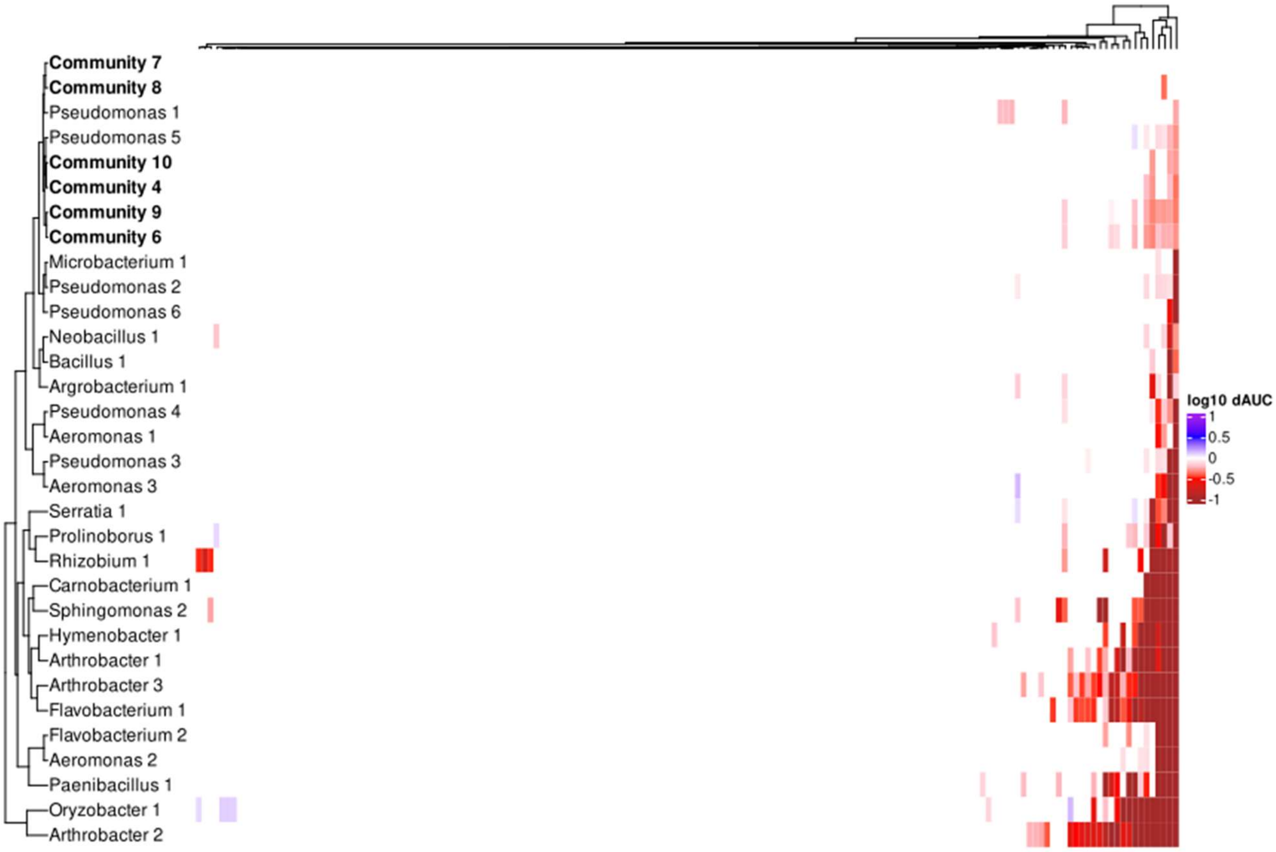

Figure S1. Heatmap of growth responses to all chemicals. Most of the 168 chemicals elicit no significant response in either the bacterial isolates or communities tested. Isolates and communities (bold) are clustered by their growth responses. Similar strains cluster together, but not perfectly by phylogeny (see Fig. 1). The communities cluster with the resilient *Pseudomonas* taxa.

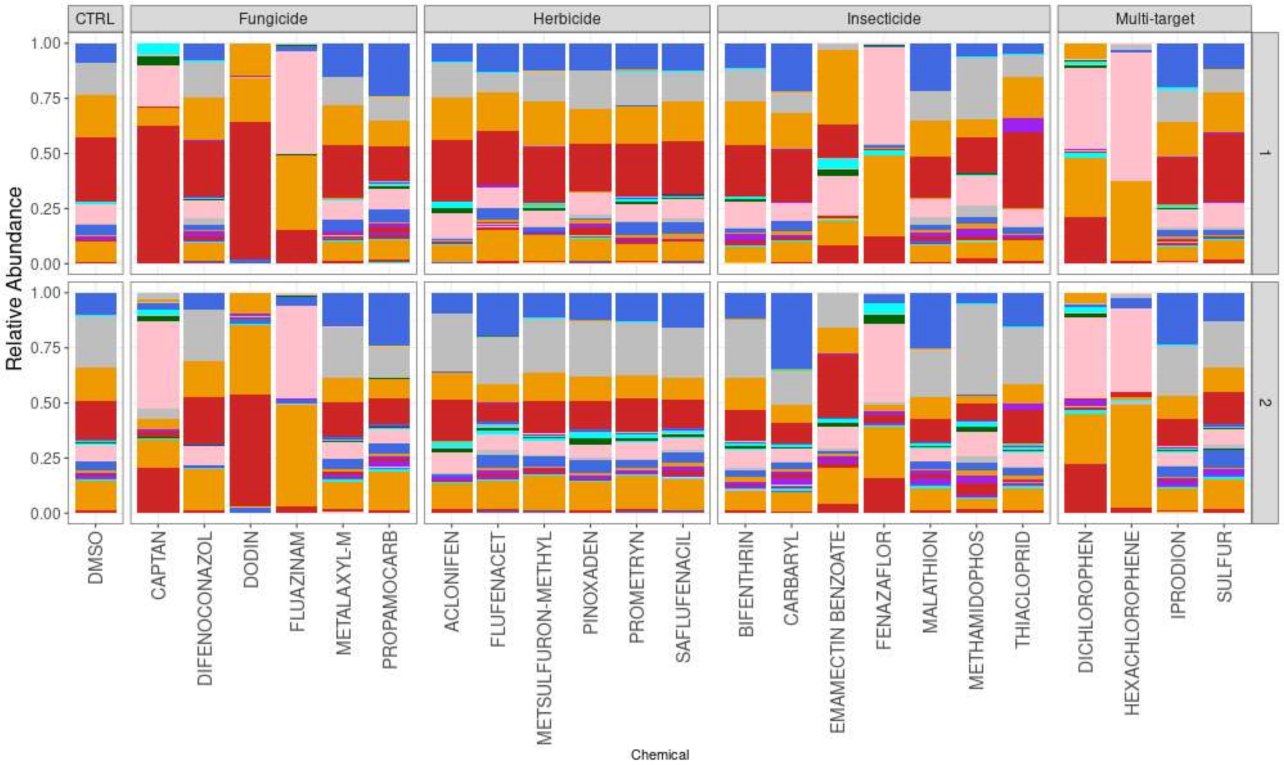

Figure S2. Species-level community relative abundance profiles. Here we show the relative abundance profiles from Community 9 using a repeating colour palette to show unique species. We can still see that, as in the aggregated genus-level abundances in Figure 1, the taxonomic profiles in the presence of most chemicals remain very similar to the DMSO control. However, the chemicals which impacted growth show distinct species-level profiles.

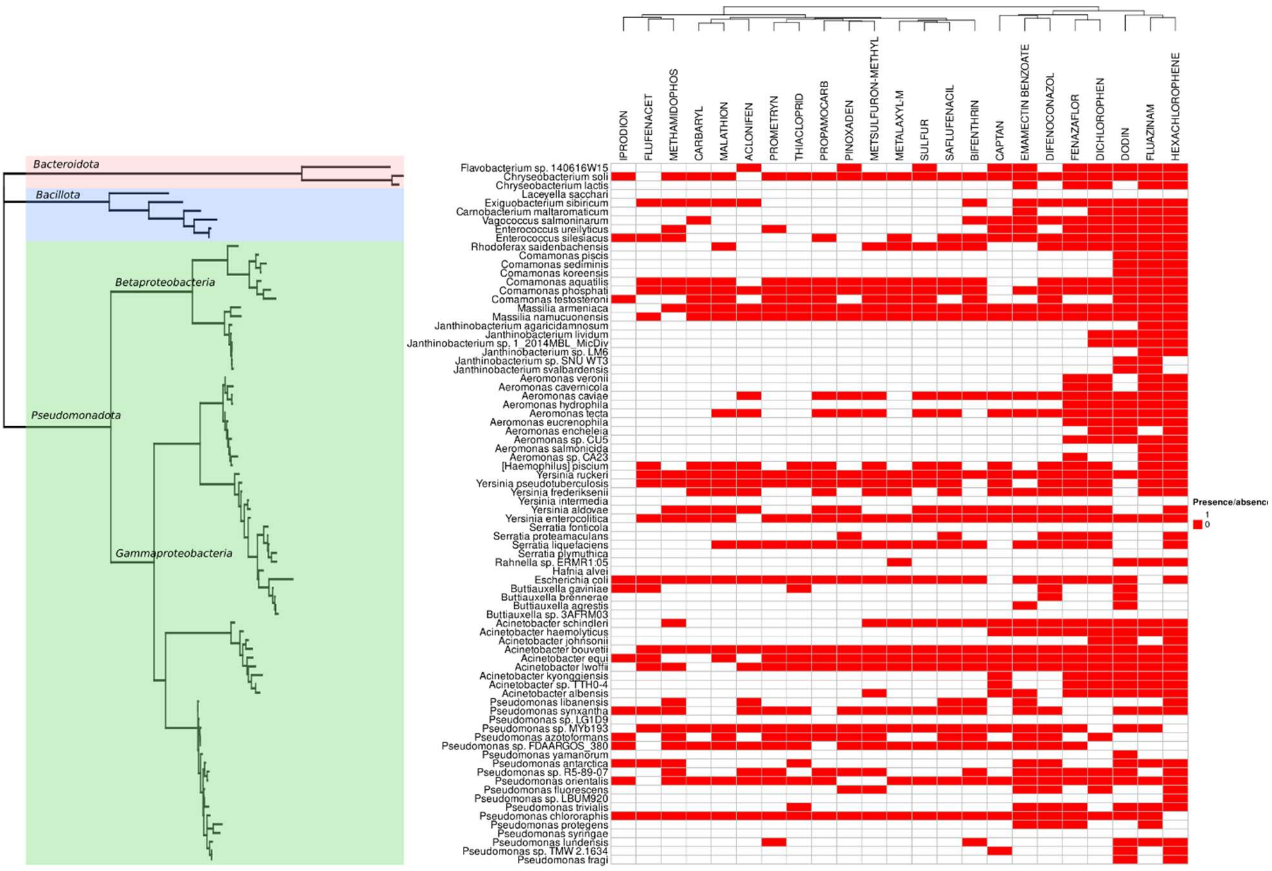

Figure S3. Phylogeny of species observed in communities grown in the presence of different chemicals. The phylogeny was built from an alignment of all species observed in our sequencing profiles. Heatmap shows presence of a species (white) or absence (red) when grown in the presence of a given chemical pollutant. Phylogeny is coloured according to the colour scheme of the isolate phylogeny (main text Fig. 2), with main branches of taxa labelled.

Supplementary Tables:

Table S1. Chemicals selected for community compositional analysis. Here we provide the chemicals and their intended targets in which community 9 was sequenced after growth in the presence of. The dAUC is the relative growth of the community compared to growth in the presence of the DMSO control (mean of 4 replicates). Asterisks denote significantly reduced growth compared to the control (Dunnett's test).

**Chemical**	**dAUC**	**Target**
ACLONIFEN	0.91	Herbicide
BIFENTHRIN	1.00	Insecticide
CAPTAN	0.53*	Fungicide
CARBARYL	0.97	Insecticide
DICHLOROPHEN	0.40*	Multi-target
DIFENOCONAZOL	1.18	Fungicide
DODIN	0.37*	Fungicide
EMAMECTIN BENZOATE	0.47*	Insecticide
FENAZAFLOR	0.43*	Insecticide
FLUAZINAM	0.43*	Fungicide
FLUFENACET	1.02	Herbicide
HEXACHLOROPHENE	0.39*	Multi-target
IPRODION	0.95	Multi-target
MALATHION	1.11	Insecticide
METALAXYL-M	1.13	Fungicide
METHAMIDOPHOS	0.62*	Insecticide
METSULFURON-METHYL	1.09	Herbicide
PINOXADEN	0.89	Herbicide
PROMETRYN	0.99	Herbicide
PROPAMOCARB	1.00	Fungicide
SAFLUFENACIL	1.12	Herbicide
SULFUR	0.95	Multi-target
THIACLOPRID	1.08	Insecticide

Table S2. Phylogenetic signal results. Test statistics, *p*-values, and adjusted *p*-values (following a Benjamini-Hochberg false discovery rate correction) for Pagel's λ and Blomberg's K, respectively. Bold values with an asterisk show significant phylogenetic signal following p-value correction ($p < 0.05$).

Chemical	λ	λ.p	λ.p.adj	K	K.p	K.p.adj
4,4-DDT	0.977	0.015	0.057	0.292	0.008	**0.036***
ABAMECTIN	0.546	<0.001	**0.008***	0.292	0.001	**0.008***
ACETOCHLOR	0.309	0.493	0.675	0.018	0.860	0.860
BIXAFEN	0.328	0.048	0.130	0.104	0.043	0.136
BROMOXYNIL	0.215	0.114	0.245	0.078	0.090	0.217
BUPROFEZIN	0.089	0.615	0.788	0.024	0.757	0.776
CAPTAN	<0.001	1.000	1.000	0.072	0.139	0.285
CARBOSULFAN	<0.001	1.000	1.000	0.030	0.648	0.718
CHLORDECON; KEPONE	0.530	0.001	**0.008***	0.381	0.001	**0.008***
CHLOROTHALONIL	0.482	0.010	**0.042***	0.213	0.003	**0.018***
CHLORPYRIPHOS-METHYL	0.266	0.029	0.092	0.099	0.104	0.237
CLOSANTEL	0.521	0.001	**0.010***	0.361	0.001	**0.008***
CYPROCONAZOL (MIXTURE OF DIASTEREOISOMER)	<0.001	1.000	1.000	0.042	0.465	0.561
CYPRODINIL	0.098	0.394	0.646	0.063	0.289	0.407
DICHLOROPHEN	0.619	0.001	**0.010***	0.178	0.003	**0.018***
DIFENOCONAZOL	0.263	0.141	0.289	0.071	0.216	0.359
DINOCAP (MIXTURE OF ISOMERS)	0.458	0.018	0.061	0.199	0.009	**0.037***
DITHIANON	0.162	0.335	0.573	0.084	0.116	0.250
DODIN	0.947	<0.001	**0.001***	0.225	0.005	**0.026***
EMAMECTIN BENZOATE	0.591	0.001	**0.010***	0.282	0.001	**0.008***
FENAZAFLOR	0.639	0.002	**0.012***	0.198	0.001	**0.008***
FENBUCONAZOL	0.232	0.165	0.322	0.059	0.267	0.407
FENHEXAMID	0.333	0.057	0.138	0.047	0.421	0.523
FLUAZINAM	0.077	0.494	0.675	0.064	0.181	0.353
FLUBENDIAMID	0.198	0.433	0.657	0.040	0.403	0.516
FLUXAPYROXAD	0.083	0.766	0.942	0.153	0.291	0.407
HEXACHLOROPHENE	0.000	1.000	1.000	0.033	0.550	0.626
IMAZALIL	0.549	0.010	**0.042***	0.157	0.016	0.060
LINURON	<0.001	1.000	1.000	0.023	0.748	0.776
METHAMIDOPHOS	0.255	0.041	0.119	0.049	0.346	0.458
MGK 264 (MIXTURE OF ISOMERS)	0.034	0.796	0.942	0.048	0.298	0.407
OXYFLUORFEN	0.203	0.453	0.663	0.056	0.280	0.407
PENTHIOPYRAD	0.434	0.057	0.138	0.140	0.072	0.197
PIPERONYL BUTOXIDE	0.026	0.804	0.942	0.038	0.545	0.626
PROCHLORAZ	0.502	0.005	**0.027***	0.109	0.058	0.170
PROFENOFOS	0.063	0.581	0.768	0.063	0.190	0.354
SPIROMESIFEN	0.243	0.099	0.225	0.093	0.219	0.359
SULFUR	0.915	0.173	0.323	0.160	0.036	0.123
TEBUCONAZOLE	0.133	0.283	0.505	0.108	0.086	0.217

TRIADIMENOL A	0.097	0.410	0.646	0.029	0.682	0.736
TRIFLOXYSTROBIN	<0.001	1.000	1.000	0.065	0.210	0.359
